# Key factors for growth and phosphate uptake by *Acinetobacter tjernbergiae*

**DOI:** 10.1007/s00253-026-13992-x

**Published:** 2026-08-05

**Authors:** Jan Brück, Simon Täuber, Leonie Ackermann, Henry Schittkowski, Peter Neubauer, Stefan Junne

**Affiliations:** 1 Chair of Bioprocess Engineering, Department of Biotechnology, Technische Universität Berlin, Ackerstraße 76 ACK24, D-13355 Berlin, Germany; 2 Department of Chemistry and Bioscience, Aalborg University Esbjerg, Niels Bohrs Vej 8, DK-6700 Esbjerg, Denmark

**Keywords:** Microbial phosphorus recovery, Polyphosphate, Overplus phenomenon, Luxury uptake, Iron supplementation

## Abstract

Phosphorus-containing fertilisers are essential for global food production but depend on finite phosphate rock reserves. Recovery of phosphorus from waste streams, such as those in wastewater treatment plants, will be required to follow future regulations. Certain *Acinetobacter* spp. can accumulate 24–30% (w w^−1^) of phosphate intracellularly, making them promising candidates for microbial phosphorus recovery. However, previous studies have reported insufficient biomass formation to enable rapid phosphate uptake. Therefore, this work investigated key factors of biomass generation and phosphate uptake of *Acinetobacter tjernbergiae* in an empirical study. Increasing iron concentration by 1 mg L^−1^ enhanced growth six-fold in shake flask cultivations. A maximum biomass concentration of 12 ± 0.7 g L^−1^ was achievable during 14 h of bioreactor cultivation. The maximum volumetric phosphate uptake rate (r_S,P_) reached 280.2 ± 40.8 mg (L h)^−1^, the highest value reported so far for *Acinetobacter* spp., while specific rates of up to 41.0 ± 1.1 mg (g h)^−1^ were achieved. It was further shown that *A. tjernbergiae* exhibits both polyphosphate accumulation and the overplus phenomenon. Moreover, phosphate uptake was strongly correlated with carbon uptake, while carbon absence resulted in phosphate release.

## Introduction

To meet the global phosphate demand, approximately 200 million metric tons of phosphate rock are mined annually (National Minerals Information Center 2024), with about 80% (w w^−1^) used for fertiliser production (Dawson and Hilton 2011; Witek-Krowiak et al. 2022). Phosphate rock, however, is a finite resource (Cordell et al. 2009; Scholz and Wellmer 2021), and its extraction has significant environmental and social consequences (Demel et al. 2018; Walsh et al. 2023). Therefore, phosphorus recovery is gaining increasing importance worldwide, driven by the global momentum towards a circular economy (Walsh et al. 2023).

In Germany, phosphorus recovery from sewage sludge exceeding 20 g phosphorus per kilogram of dry matter will become mandatory by 2029 for wastewater treatment plants (WWTPs) with over 100,000 population equivalents (Sichler et al. 2022). Yet projections suggest that the recovery capacities will remain insufficient by then, as only a few among the many technologies have reached successful large-scale implementation (FiW 2025; ESPP 2026). Uniform applicability is further hindered by the wide variation in WWTPs operating conditions (Egle et al. 2015; Guo et al. 2026) and inconsistent sewage sludge composition (Sichler et al. 2022).

Moreover, the presence of heavy metals, organic pollutants, and pathogens that prevent direct application of sewage sludge as a fertiliser must be addressed within the recovery process (Xu et al. 2023; Guo et al. 2026). The development and optimisation of cost-effective and environmentally friendly recovery methods from WWTP process streams and sewage sludge, thus, remains a challenge (Egle et al. 2015; Xu et al. 2023; Guo et al. 2026). The utilisation of microorganisms that are capable of storing large amounts of polyphosphate (polyP) intracellularly presents a biological recovery method that might be able to address these challenges (Yang et al. 2017; Vučić and Müller 2021; Vučić et al. 2024). Aqueous side streams generated during sludge treatment can represent suitable targets for biological phosphorus recovery. At a German WWTP, the release of phosphorus from water separated from sludge contained 51–106 mg L⁻^1^ of free phosphorus (Vučić et al. 2024). Enhanced biological phosphorus removal promotes release into the supernatant during sludge digestion, whereas iron-based separation binds P in solid form as vivianite (Egle et al. 2016; Heinrich et al. 2023). PolyP accumulation has been documented in numerous microorganisms, positioning them as promising candidates for phosphorus recovery (Vučić et al. 2024). Phosphorus in structural cellular components typically comprises 1–2% (w w^−1^) of dry biomass (Blackall et al. 2002; Yang et al. 2017). PolyP accumulation allows far higher intracellular storage, with maximum phosphorus contents reaching up to 4–6% for microalgae (Schaedig et al. 2023; Solovchenko et al. 2024), 3–5% for *Escherichia coli* (Kato et al. 1993), and 7–8% (all w w^−1^) for *Saccharomyces cerevisiae* (Christ and Blank 2019; Vučić et al. 2024) in natural isolates. In non-axenic cultures enriched with phosphate-accumulating organisms (PAOs), a phosphorus content of 8–15% (w w^−1^) has been measured in activated sludge (Blackall et al. 2002; Yang et al. 2017). Intracellular phosphorus contents of 8–10% (w w^−1^) in axenic cultures of *Acinetobacter* spp. are unique among natural isolates (Deinema et al. 1980; Van Groenestijn et al. 1989), and together with their prevalence in WWTPs, led to their extensive investigation between the 1980s and early 2000s in the context of biological phosphorus removal (EBPR) (Stokholm-Bjerregaard et al. 2017).

Interest declined once it became apparent that *Acinetobacter* spp. do not exhibit the characteristic anaerobic phosphate release and aerobic phosphate uptake behaviour of canonical PAOs (Tandoi et al. 1998; Seviour et al. 2003; Ruiz-Haddad et al. 2024). While research has provided valuable insights into the mechanisms and environmental conditions influencing polyP accumulation in *Acinetobacter* spp., comparatively little effort has been made to investigate how to increase biomass concentrations, a key factor for rapid phosphate uptake.

Studies reporting on optimising the growth of *Acinetobacter* spp. isolated from WWTPs identified 30 °C and pH 7.5 as suitable conditions for the growth of most strains (Hao and Chang 1987; Van Groenestijn et al. 1989; Han et al. 2018; Chen et al. 2021). A molar carbon-to-nitrogen ratio of 8–10 appears advantageous, particularly when using acetate as carbon and ammonium as nitrogen sources (Pauli and Kaitala 1995; Han et al. 2018; Chen et al. 2021). This aligns with most *Acinetobacter* spp., which do not require exogenous vitamins or amino acids for their growth (Warskow and Juni 1972). Sufficient supplementation with calcium, potassium, and magnesium is critical (Van Groenestijn et al. 1988; Pauli and Kaitala 1995), while studies on some strains report additional growth benefits from iron addition (Gentile et al. 2014; Sheldon and Skaar 2020).

The biochemistry of polyP storage in *Acinetobacter* spp. is extensively documented. It primarily accumulates polyP intracellularly as linear chains of orthophosphate residues linked by high-energy phosphoanhydride bonds (Neville et al. 2022), typically forming dense cytoplasmic granules (Bark et al. 1992; Weltin et al. 1996). The polyP metabolism is tightly coupled to cellular energy metabolism and aerobic respiration in *Acinetobacter* spp. (Van Veen et al. 1993). Whenever energy generation exceeds the biosynthetic demand while phosphate is available in excess, cells accumulate polyP as a combined energy and phosphorus reservoir, a process commonly referred to as luxury phosphate uptake (Van Groenestijn 1988; Ghigliazza et al. 1998). In contrast, phosphate release is typically observed under unfavourable conditions such as carbon starvation or anaerobiosis, where polyP degradation contributes to intracellular ATP regeneration (Ohtake et al. 1985; Van Groenestijn and Deinema 1987).

Polyphosphate kinases primarily mediate the synthesis and degradation of polyP. Polyphosphate kinase 1 (PPK1) catalyses the ATP-dependent elongation of polyP chains (Trelstad et al. 1999), whereas polyphosphate kinase 2 (PPK2) catalyses polyP-dependent phosphorylation of AMP to ADP (Itoh and Shiba 2004). Depending on intracellular conditions, both enzymes may also exhibit reversible activity (Itoh and Shiba 2004). Although not directly involved in polyP synthesis or degradation, the enzyme adenylate kinase (AK1) supports its function, maintaining the balance among intracellular AMP, ADP, and ATP pools, thereby linking polyP turnover to cellular energy homeostasis (Resnick and Zehnder 2000).

Several environmental and nutritional factors influence polyP accumulation in *Acinetobacter* spp. Particularly, high phosphate assimilation rates are frequently observed when cells are transferred from phosphate-limited to phosphate-rich conditions, a response known as the overplus phenomenon (Ohtake et al. 1985; Pauli and Kaitala 1997; Van Niel et al. 1999). In addition, the limitation of other essential nutrients, including carbon and sulphur, has been reported to enhance intracellular polyP accumulation, suggesting that imminent energy or nutrient depletion represents a general trigger for polyP storage (Ohtake et al. 1985; Van Groenestijn et al. 1989).

Metal ions also play a critical role in polyP metabolism and storage. Uptake and release of phosphate have been shown to occur in close temporal association with magnesium and calcium ions (Van Groenestijn et al. 1988; Van Veen et al. 1994). Magnesium is considered the primary counterion required for polyP synthesis and stabilisation (Van Groenestijn et al. 1988; Shiba et al. 2005), while calcium can partially substitute under magnesium-limited conditions (Van Groenestijn et al. 1988).

Despite extensive research on polyP accumulation by *Acinetobacter* spp., the maximum biomass concentration reported in the literature has, to our knowledge, not exceeded 4 g L^−1^ yet. This limits research and applicability for phosphorus recovery processes (Srivastava and Srivastava 2005). The present study aims to identify key factors that increase the growth rate and biomass titre of aerobic *A. tjernbergiae* cultures within an empirical approach, with the main objective of increasing volumetric phosphate uptake. By investigating medium composition and feeding strategies in shake flask and fed-batch bioreactor experiments, this work provides new insights into the importance of key growth factors and the coupling between growth state and phosphate uptake in *A. tjernbergiae* cultures.

## Materials and methods

### Strain and maintenance

The strain *A. tjernbergiae* 7N16 was obtained from the German Collection of Microorganisms and Cell Cultures (DSMZ, Braunschweig, Germany). Cells were preserved in cryogenic vials containing 50% (w w^−1^) glycerol and 50% (w w^−1^) preculture medium and stored at − 80 °C. A working cell bank was maintained on agar plates, which were re-plated every 2 weeks to ensure culture viability. Plates were stored at 4 °C. Throughout all experiments, cultures were routinely examined for contamination using light microscopy at 1000 × magnification.

### Culture media and feed solutions

The preculture medium was composed of (in g L^−1^) 15 tryptone (peptone from casein), 5 yeast extract (Ohly CPT, Hamburg, Germany), 5 malt extract, 2 glycerol (≥ 99.5%), and 1 MgSO_4_ × 7 H_2_O (≥ 99%). Unless stated otherwise, all components were obtained from Carl Roth (Karlsruhe, Germany). For agar plate preparation, 20 g L^−1^ agar–agar (Kobe I, Carl Roth) was added to the respective medium.

A culture medium with reduced phosphate concentration, hereafter referred to as PR medium (phosphate-reduced medium), and modifications thereof were used in all experiments. It was composed as follows (in g L^−1^): 1 tryptone, 0.1 yeast extract (Ohly CPT), 0.5 MgSO_4_ × 7 H_2_O (≥ 99%), 0.015 CaCl_2_ × 2 H_2_O (≥ 99%), 0.92 K_2_HPO_4_ (≥ 99%), 0.87 NH_4_Cl (≥ 99.7%), 6.96 C_2_H_3_NaO_2_ (Honeywell, Seelze, Germany). Furthermore, 1 mL supplementary mineral salt solution was added per litre. This consisted of (in g L^−1^) 1.2 MnCl_2_ × 4 H_2_O, 0.4 FeSO_4_ × 7 H_2_O, 0.3 CoCl_2_ × 6 H_2_0, 0.2 ZnSO_4_ × 7 H_2_O, 0.2 Na_2_MoO_4_ × 2 H_2_O (Merck, Darmstadt, Germany), 0.1 CaCl_2_ × 2 H_2_O, 0.02 CuSO_4_ × 5 H_2_O (VWR, Leuven, Belgium), and 0.02 NiCl_2_ × 6 H_2_O (Merck). Adjustments to the medium are mentioned in the respective experiments. Additional iron and zinc were supplied as FeSO₄ × 7 H₂O and ZnSO₄ × 7 H₂O, respectively (both VWR).

OrganicN medium is a major modification of the PR medium, in which all NH_4_Cl was replaced by yeast extract and tryptone. It contained (in g L^−1^) 1.6 tryptone, 1.5 yeast extract (Ohly CPT), 0.5 MgSO_4_ × 7 H_2_O (≥ 99%), 0.015 CaCl_2_ × 2 H_2_O (≥ 99%), 0.205 K_2_HPO_4_ (≥ 99%), 6.96 C_2_H_3_NaO_2_ (Honeywell), an additional 0.02 ZnSO₄ × 7 H₂O (VWR), and 0.01 FeSO₄ × 7 H₂O (VWR) together with 1 mL L^−1^ mineral salt solution.

The feed solutions used in bioreactor experiments contained C_2_H_3_NaO_2_ (Honeywell), NH_4_Cl (Carl Roth), KH_2_PO_4_ (Thermo Fisher Scientific, Loughborough, UK), and FeSO₄ × 7 H₂O (VWR). The specific concentrations and corresponding feeding strategies for each experiment are provided below.

### Preculture preparation

Depending on the experiment, precultures were prepared in either 125 mL or 250 mL baffled shake flasks (Ultra Yield® Flasks, Thomson Instrument Company, Carlsbad, CA). For shake flask experiments, 25 mL of preculture medium was added to the 125 mL flasks, whereas 50 mL of preculture medium was added to 250 mL flasks for bioreactor inoculations. All precultures were initiated from single *A. tjernbergiae* colonies obtained from the working cell bank. Each colony was first transferred to 2 mL of preculture medium in a 15 mL centrifuge tube and homogenised by quickly vortexing. The resulting suspension was subsequently used to inoculate flasks, which were sealed with gas-permeable membrane caps (AirOtop®, Thomson Instrument Company). Cultivation was carried out on an orbital shaker (Multitron Standard, Infors HT, Bottmingen, Switzerland) at 30 °C and 250 rpm with an amplitude of 50 mm until an optical density (OD₆₀₀) of 4 was reached.

### Main shake flask cultures

Shake flask experiments were conducted in duplicate using 250 mL Ultra Yield® flasks equipped with AirOtop® seals, and with a working volume of 50 mL. Cultures were inoculated to an initial OD₆₀₀ of 0.5. To minimise carryover effects from the preculture medium, the inoculum was centrifuged at 20,000 × *g* for 5 min at 4 °C (5810 R, Eppendorf, Hamburg, Germany), the supernatant was separated, and the cells were resuspended in culture medium and then transferred to the main culture.

### Bioreactor cultivations

Bioreactor cultivations were conducted in duplicate using a 1 L MultiFors® bioreactor system (Infors HT) operated at 30 °C and a pH value of 7.5. Process control and data acquisition were performed using the integrated control unit and the Eve® software version 2025 (Infors HT). All experiments started with a total working volume of 500 mL, consisting of 450 mL culture medium and 50 mL inoculum, corresponding to an initial OD₆₀₀ of 0.4.

*Online* monitoring was conducted with a pH probe (EasyFerm Plus PHI Arc 225, Hamilton Bonaduz, Switzerland) and a dissolved oxygen probe (VisiFerm DO Arc 225 H2, Hamilton). pH was controlled using 2 M sulphuric acid (H₂SO₄, VWR). Dissolved oxygen was kept at 40% of saturation using a cascade control. Aeration rates were manually adjusted as needed. Antifoam (Antifoam 204, Sigma-Aldrich, Steinheim, Germany) was added manually whenever required. Sampling was performed at defined intervals throughout the batch and feeding phases.

Both bioreactor experiments consisted of an initial batch phase followed by an exponential feeding phase, during which the feed rate was set according to a specific growth rate of *μ* = 0.3 h^−1^. The exponential feed solution employed during experiments with phosphate pulses was prepared with the following components (in g L^−1^): 104 C_2_H_3_NaO_2_, 20.7 NH_4_Cl, 3.43 KH_2_PO_4_, and 0.3 FeSO₄ × 7H₂O, corresponding to a molar C:N:P ratio of 100:15:1. The initial feed rate was 13 mL h^−1^, with a total added volume of 100 mL. The two phosphate pulse solutions contained (in g L^−1^) 173.63 C_2_H_3_NaO_2_, 11 NH_4_Cl, and 57.24 KH_2_PO_4_, resulting in a C:N:P ratio of 10:0.5:1; 12 mL was added per pulse. The first pulse was added immediately after the end of the exponential feeding, and the second pulse 2 h later. During this time, the sampling frequency was increased to 30 min intervals. The feed solution with modified phosphate concentration contained (in g L^−1^) 125.01 C_2_H_3_NaO_2_, 24.84 NH_4_Cl, 8.31 KH_2_PO_4_, and 0.375 FeSO_4_ × 7 H_2_O, corresponding to a molar C:N:P ratio of 100:15:2. The initial feed rate was set to 10 mL h^−1^, with a total added volume of 200 mL.

### Growth analysis

Optical density was measured at 600 nm using disposable cuvettes and a spectrophotometer (Ultrospec 2,100 Pro, Amersham plc, UK). Samples were diluted with 0.9% (w w^−1^) NaCl solution to remain within the linear measurement range. Cell dry weight (CDW) was determined in duplicate by centrifuging 1 mL of culture at 21,500 × *g* for 10 min at 4 °C (CT15RE, VWR). The supernatant was filtered through a 0.2-μm PTFE filter before storage and analysis. The cell pellet was washed once with 0.9% NaCl, then dried at 80 °C for 24 h before the vessel’s weight difference was determined with an analytical balance (BP210S, Sartorius, Göttingen, Germany). *Offline* pH measurements were performed using a calibrated pH meter (Fe20, Mettler Toledo, Urdorf, Switzerland).

### Acetate quantification

Acetate concentrations were determined by HPLC equipped with a refractive index detector (1,200 Series, Agilent Technologies, Waldbronn, Germany) using an H⁺ 8 µm column (Hyper REZ XP, Thermo Fisher Scientific, Waltham, MA). The mobile phase consisted of 5 mM H₂SO₄ at a flow rate of 0.6 mL min^−1^ and a column temperature of 65 °C. 2-Butanone (2 g L^−1^) was used as an internal standard. Samples exceeding 10 g L^−1^ acetate were diluted accordingly.

### Phosphate quantification

Orthophosphate concentrations were quantified using a modified molybdenum blue method based on Saheki et al. (1985). Samples were acidified with 1 M HCl at 95 °C for 20 min to release bound phosphate before analysis. Colour development was achieved via a zinc-molybdate complex formation followed by reduction with ascorbic acid. Absorbance was measured at 850 nm, and concentrations were calculated using KH₂PO₄ calibration standards.

### Data analysis and visualisation

Data analysis was performed using Microsoft Excel, and graphs were generated using Origin (Origin 2025b, OriginLab, Northampton, MA). Values represent means of duplicate experiments with standard deviations. Reactor volumes were corrected for feed addition and sampling. Specific growth rates, substrate uptake rates, and yields were calculated according to the equations provided below.

1 $$\mu=\frac{ln{(X}_2V_2)-ln{(X}_1V_1)}{t_2-t_1}$$

2 $${q}_{S,i}=-\frac{1}{{XV}}\times \frac{\Delta ({S}_{i}V)}{{t}_{2}-{t}_{1}}$$

3 $${r}_{S,i}={q}_{S,i} \times X$$

4 $${Y}_{XS,i}=\frac{\Delta (XV)}{\Delta ({S}_{i}V)}$$

5 $${Y}_{PS,C}=\frac{\Delta \left(PV\right)}{\Delta ({S}_{C}V)}$$

## Results

This study aimed to assess the volumetric and specific phosphate assimilation capacity of *A. tjernbergiae* by investigating the influence of media composition and cultivation mode on biomass yield and phosphate uptake capacity in shake flask and bioreactor cultivations.

### Influence of iron and zinc

Since most *Acinetobacter* spp. are prototrophic and efficiently utilise acetate and ammonium as carbon and nitrogen sources, insufficient availability of trace metals was hypothesised. We therefore tested iron and zinc supplementation, increasing their concentrations 26- and 101-fold in the cultivation medium.

Additional iron (10 mg L^−1^ FeSO_4_ × 7H_2_O) and zinc (20 mg L^−1^ ZnSO_4_ × 7H_2_O) were supplemented to the PR medium, described as PR + FeZn medium in the following. These specific iron and zinc concentrations were selected, as it is reported that both support the growth of the polyP-accumulating bacterium *Pseudomonas stutzeri* in asparagine minimal medium (Xie et al. 2014; Sommer et al. 2017).

**Fig. 1:**
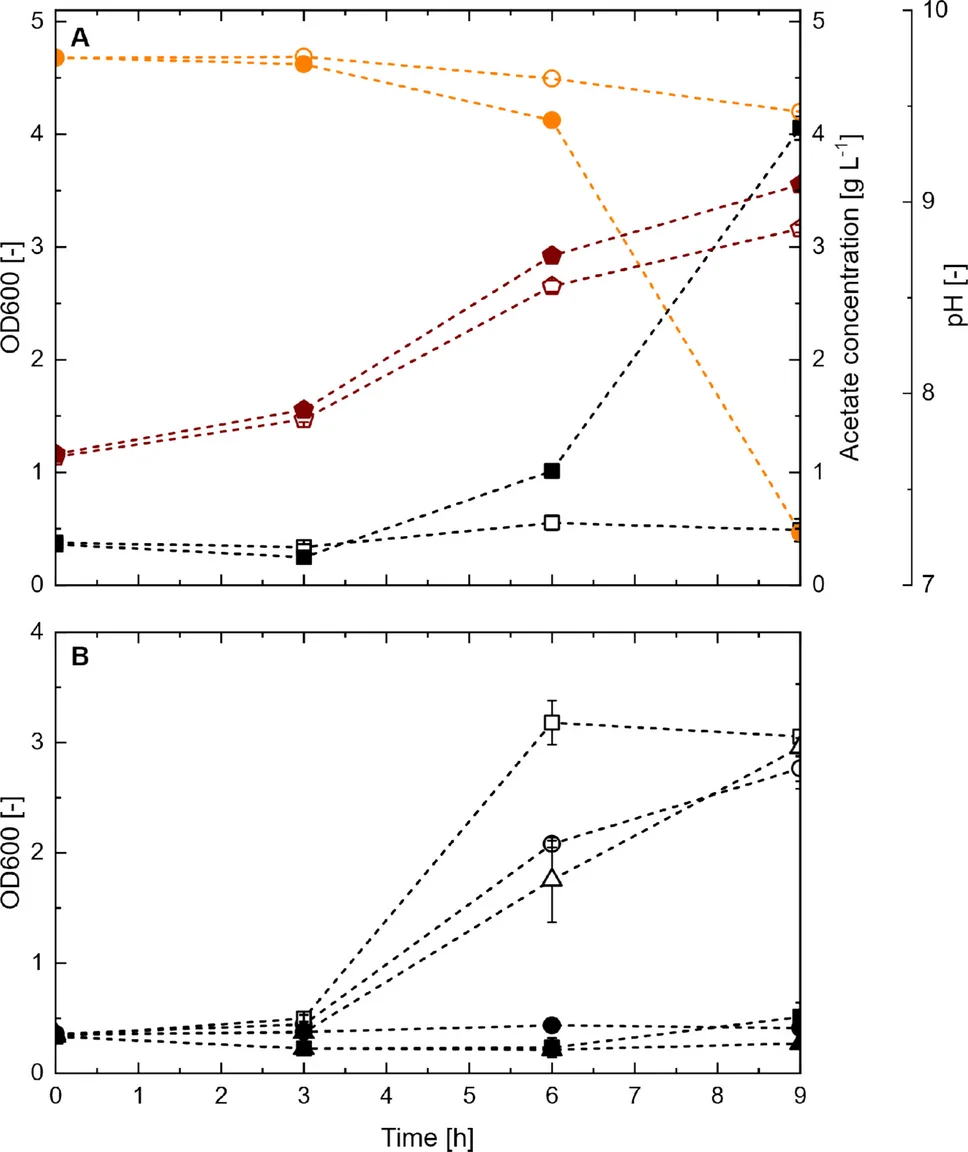
Effect of iron and zinc supplementation on growth in shake flask cultures of *A. tjernbergiae*. **A** Growth in PR medium is depicted by the empty shapes. Growth in PR + FeZn medium containing supplementary iron (2.1 mg L^−1^, + 26-fold) and zinc (4.6 mg L^−1^, + 101-fold) is depicted by filled shapes, OD_600_ (black squares), acetate concentration (orange circles), and pH (red pentagons) in duplicate cultures. **B** Effect of varying iron or zinc additions on growth in PR-NH_4_-PO_4_ medium (reduction of ammonia concentration). Iron concentrations are depicted by empty shapes: circles 1.1 mg L^−1^ (+ 13.5-fold), squares 4.1 mg L^−1^ (+ 51-fold), triangles 8.1 mg L^−1^ (+ 101-fold). Zinc concentrations are depicted by filled shapes: circles 2.3 mg L^−1^ (+ 51-fold), squares 9.1 mg L^−1^ (+ 201-fold), triangles 18.2 mg L^−1^ (+ 401-fold). Error bars indicate ± SD

Cultures grown in the PR + FeZn medium reached a final OD₆₀₀ of 4.1 ± 0.1, corresponding to an eight-fold increase in comparison to the PR medium (Fig. 1A). During exponential growth between 3 and 9 h, a maximum specific growth rate of *μ*_max_ = 0.47 ± 0.00 h^−1^ was determined in PR + FeZn medium. This growth was accompanied by rapid acetate consumption, with concentrations decreasing from 4.6 ± 0.1 g L^−1^ to 0.34 ± 0.13 g L^−1^. In contrast, acetate concentrations remained largely unchanged in the PR medium. The pH increased similarly in both media, from approximately 7.7 to final values of 8.8 and 9.1, respectively. Therefore, it is unlikely that the pH increase was dominantly caused by acetate consumption, but rather by the assimilation of amino acids from tryptone, and from hydrogen and dihydrogen phosphate.

To further identify which metal ion was responsible for the increase in growth rate, iron and zinc concentrations were changed independently. In comparison to the previous experiment, the PR medium was modified by decreasing concentrations of K_2_HPO_4_ to 0.28 g L^−1^ and of NH_4_Cl to 0.5 g L^−1^ to reduce the increase in the pH value at the end of the cultivation. This modified medium, hereafter denoted PR-NH_4_-PO_4_, was supplemented with (in mg L^−1^) either 5, 20, or 40 FeSO_4_ × 7 H_2_O, or 10, 40, or 80 ZnSO_4_ × 7 H_2_O.

Supplementation with iron resulted in a clear increase in biomass formation (Fig. 1B). Final OD₆₀₀ values of approximately 3 were reached within 6 to 9 h of cultivation. The addition of 40 mg L^−1^ FeSO₄ × 7 H₂O resulted in the highest OD₆₀₀ of 3.2 ± 0.2, while concentrations of 1.1 mg L^−1^ yielded OD₆₀₀ values of 2.8 ± 0.1. The specific growth rate *μ*_max_ was 0.52 ± 0.09 h^−1^, 0.62 ± 0.00 h^−1^, and 0.51 ± 0.06 h^−1^ at increasing iron concentrations from 1.1 to 8.1 mg L^−1^. In contrast, zinc supplementation did not result in any increase in the OD₆₀₀, confirming iron to be the primary growth limitation in PR medium. As the basal PR medium already contained iron in the range of trace metal supplementation typically used in defined mineral media, the growth response indicates an unusually high iron requirement of *A. tjernbergiae*. The iron concentrations ranged from 1.1 to 8.1 mg L⁻^1^ elemental iron, a 13.5- to 101-fold increase compared to the PR medium. These concentrations are considerably above standard levels in typical bacterial growth media.

### Influence of the nitrogen source

To test whether complex nitrogen sources would further enhance the growth of *A. tjernbergiae*, ammonium was replaced with yeast extract and tryptone. In the medium described as OrganicN medium in the following, all 0.87 g L^−1^ NH_4_Cl was replaced with 0.6 g L^−1^ tryptone (13%-w w^−1^ N) and 1.4 g L^−1^ yeast extract (10%-w w^−1^ N). These concentrations were chosen so that the total nitrogen concentration remained approximately equivalent to that in the PR medium. The K₂HPO₄ concentration was further reduced to 0.205 g L^−1^ to compensate for the approximate phosphorus intake by the yeast extract and tryptone in the OrganicN medium.

A new reference medium, hereafter denoted PR + FeZn-PO_4_, was a modification of the PR medium in which the K_2_HPO_4_ concentration was reduced to 0.28 g L^−1^ and FeSO_4_ × 7 H_2_O and ZnSO_4_ × 7 H_2_O were added at concentrations of 10 mg L^−1^ and 20 mg L^−1^, respectively. Although no zinc limitation had been identified, the results indicated no negative effect of zinc supplementation. To ensure an excess of zinc, its concentration was therefore increased in parallel with the iron concentration. The phosphate concentration matched that in the PR-NH_4_-PO_4_ medium, and the iron and zinc additions were the same as in PR + FeZn medium.

**Fig. 2:**
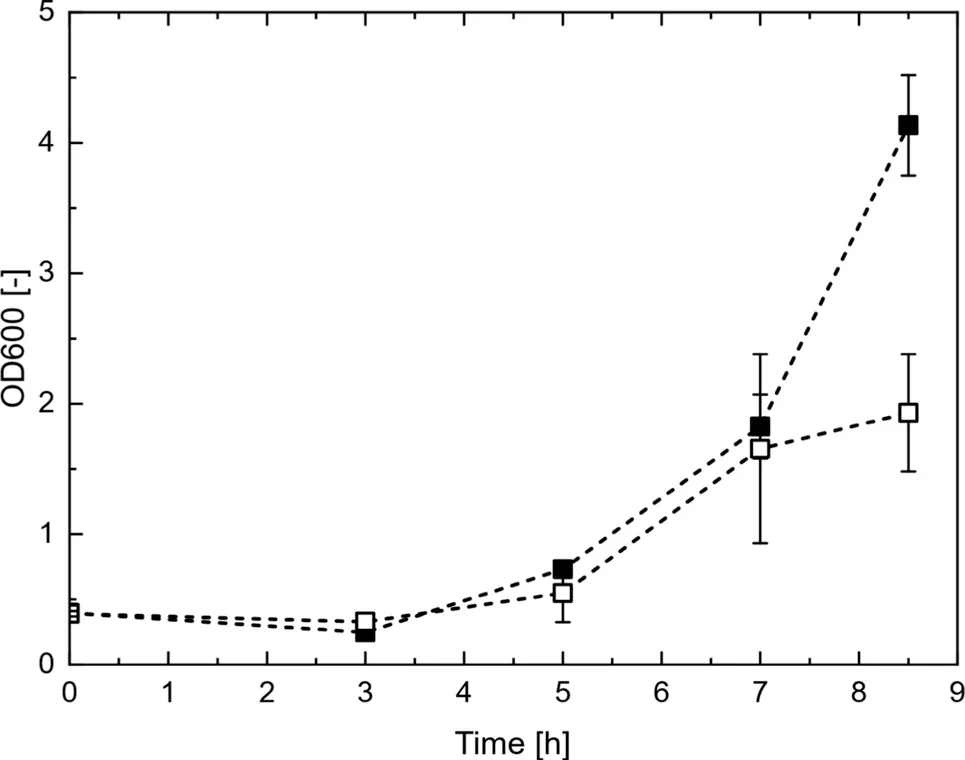
Influence of the nitrogen source on the growth of *A. tjernbergiae* in shake flask cultivations. OD values of cultures grown in PR + FeZn-PO_4_ medium (filled squares) and in OrganicN medium (empty squares). Both media have the same composition, but the entire NH_4_Cl in the PR + FeZn-PO_4_ medium is replaced with tryptone and yeast extract in OrganicN medium. The error bars indicate ± SD

Maximum growth rates were comparable between the PR + FeZn-PO_4_ and the OrganicN medium, with values of 0.55 ± 0.03 h^−1^ and 0.54 ± 0.02 h^−1^, respectively. However, biomass accumulation ceased after around 7 h in OrganicN medium, yielding a significantly lower final OD₆₀₀ of 1.9 ± 0.5 in comparison to the OD₆₀₀ of 4.1 ± 0.4 in the PR + FeZn-PO_4_ medium (Fig. 2). These results confirm that ammonium addition is suitable for maximal growth of *A. tjernbergiae*, with complex nitrogen sources providing no additional benefits for achieving higher growth rates.

### Bioreactor cultivations

Growth and phosphate uptake of *A. tjernbergiae* were investigated further in pH-controlled bioreactor cultivations. Besides the previously applied batch mode in shake flasks, this time, both batch and carbon-limited fed-batch conditions were applied. It was investigated whether different C:P and C:N:P ratios affected phosphate uptake.

Growth was observed during exponential feeding of defined components using a *μ*_set_ of 0.3 h^−1^ and a C:P ratio of 100:1. The culture medium was an alteration of the previously used PR medium with the following adjustments (in g L^−1^): 0.28 K_2_HPO_4_, 1 MgSO_4_ × 7 H_2_O, 0.03 CaCl_2_ × 2 H_2_O, and 2 mL L^−1^ mineral salt solution to account for the prolonged cultivation time, and an extra addition of FeSO_4_ × 7 H_2_O and ZnSO_4_ × 7 H_2_O. In the medium, both iron (4.1 mg L^−1^) and zinc concentrations (2.3 mg L^−1^) were increased by about 51-fold compared to the PR medium (denoted as PR-OPT in the following) to ensure that both are present in surplus. The exponential feed solution consisted of (in g L^−1^) 104 C₂H₃NaO₂, 20.7 NH₄Cl, 3.43 KH₂PO₄, and 0.3 FeSO₄ × 7 H₂O, resulting in a C:N:P ratio of 100:15:1. To further determine whether phosphate uptake by *A. tjernbergiae* is affected by low carbon availability, two pulses with high phosphate but low acetate concentrations were added at the end of the exponential feeding phase. The feed of the two phosphate pulses had a C:N:P ratio of 10:0.5:1. The first pulse was added upon acetate depletion, and the second pulse was added exactly 2 h later.

**Fig. 3:**
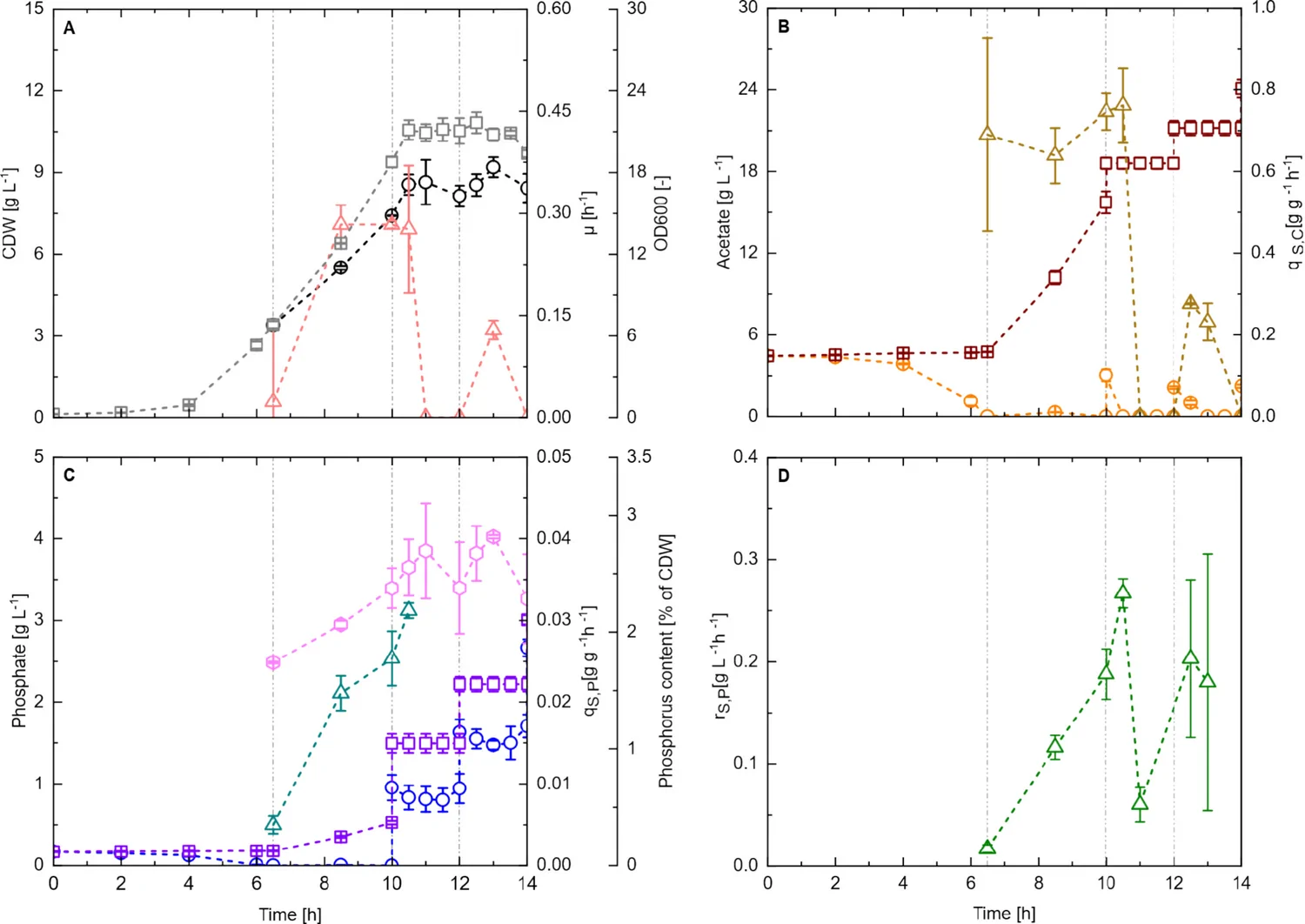
Batch/fed-batch bioreactor cultivations of *A. tjernbergiae*. The start of the exponential feeding (*μ*_set_ = 0.3 h^−1^) is marked with a dotted line at 6.5 h. Phosphate pulse additions are marked with dotted lines at 10 h and 12 h. The first pulse addition was applied after acetate depletion, the second 2 h later. Cultivations were done in duplicates for 14 h. The error bars indicate ± SD. **A** CDW (black circles), specific growth rate *μ* (red triangles), and optical density OD_600_ (grey squares). **B** Acetate concentration (orange circles), total acetate addition (red squares), and specific acetate uptake rate (golden triangles). **C** Phosphate concentration (blue circles), total phosphate addition (violet squares), specific phosphate uptake rate (cyan triangles), and specific phosphorus content (pink circles). **D** Volumetric phosphate uptake rate

During cultivation in the PR-OPT medium, *A. tjernbergiae* displayed typical growth behaviour in batch mode, with a short lag phase followed by rapid acetate and phosphate consumption and concurrent biomass accumulation (Fig. 3). A maximum specific growth rate *μ*_max_ = 0.49 ± 0.01 h^−1^ was determined during this period. During the exponential feeding phase, the CDW increased from 3.4 ± 0.1 to 7.4 ± 0.0 g L^−1^ with an average specific growth rate of *μ* = 0.28 ± 0.00 h^−1^. The specific acetate uptake rate q_S,C_ reached 0.63 ± 0.03 g (g h)^−1^ during this phase, while 22.5 ± 2.7 mg (g h)^−1^ were determined for the specific phosphate uptake rate (q_S,P_). The biomass yields on acetate (Y_XS,C_) and phosphate (Y_XS,P_) were 0.39 ± 0.03 g g^−1^ and 11.3 ± 1.3 g g^−1^, respectively (Table 2), leading to a phosphate uptake yield on acetate of Y_PS_C = 35.0 ± 1.5 mg g^−1^.

Following the first phosphate pulse, q_S,P_ increased to a maximum of 31.2 ± 0.9 mg (g h)^−1^, accompanied by an increase in CDW to 8.6 ± 0.4 g L^−1^ and specific phosphorus content of 2.7 ± 0.4% (w w^−1^). The volumetric phosphate uptake rate (r_S,P_) peaked at 266.9 ± 14.2 mg (L h)^−1^.

These results demonstrate that the exponential feeding regime led to a comparably high growth rate for several hours. The C:P ratio of 100:1 caused almost phosphate depletion, with concentrations below 3 mg L^−1^. The first pulse increased q_S,P_ when excess phosphate and acetate were available, while the uptake declined under acetate-limiting conditions.

### Feeding with increased phosphate content

Following the previous experiment’s demonstration of sustained high growth rates through exponential feeding, this cultivation extended the feeding phase to 6.5 h to determine the maximum achievable biomass. Additionally, since a molar C:P ratio of 100:1 led to almost phosphate depletion, the ratio was changed to 100:2 to challenge phosphate uptake.

The culture medium was another alteration of the PR medium as follows (in g L^−1^): 4.65 C_2_H_3_NaO_2_, 0.59 NH_4_Cl, 0.19 K_2_HPO_4_, 0.67 tryptone, 0.067 yeast extract, 1 MgSO_4_ × 7 H_2_O, 0.03 CaCl_2_ × 2 H_2_O, an extra addition of 0.02 FeSO_4_ × 7 H_2_O and 0.01 ZnSO_4_ × 7 H_2_O beside 2 mL L^−1^ of the mineral salt solution. This represents a reduction of carbon, nitrogen, and phosphate in the base medium by one-third to clearly examine the effects of changes in the feed solution composition.

**Fig. 4:**
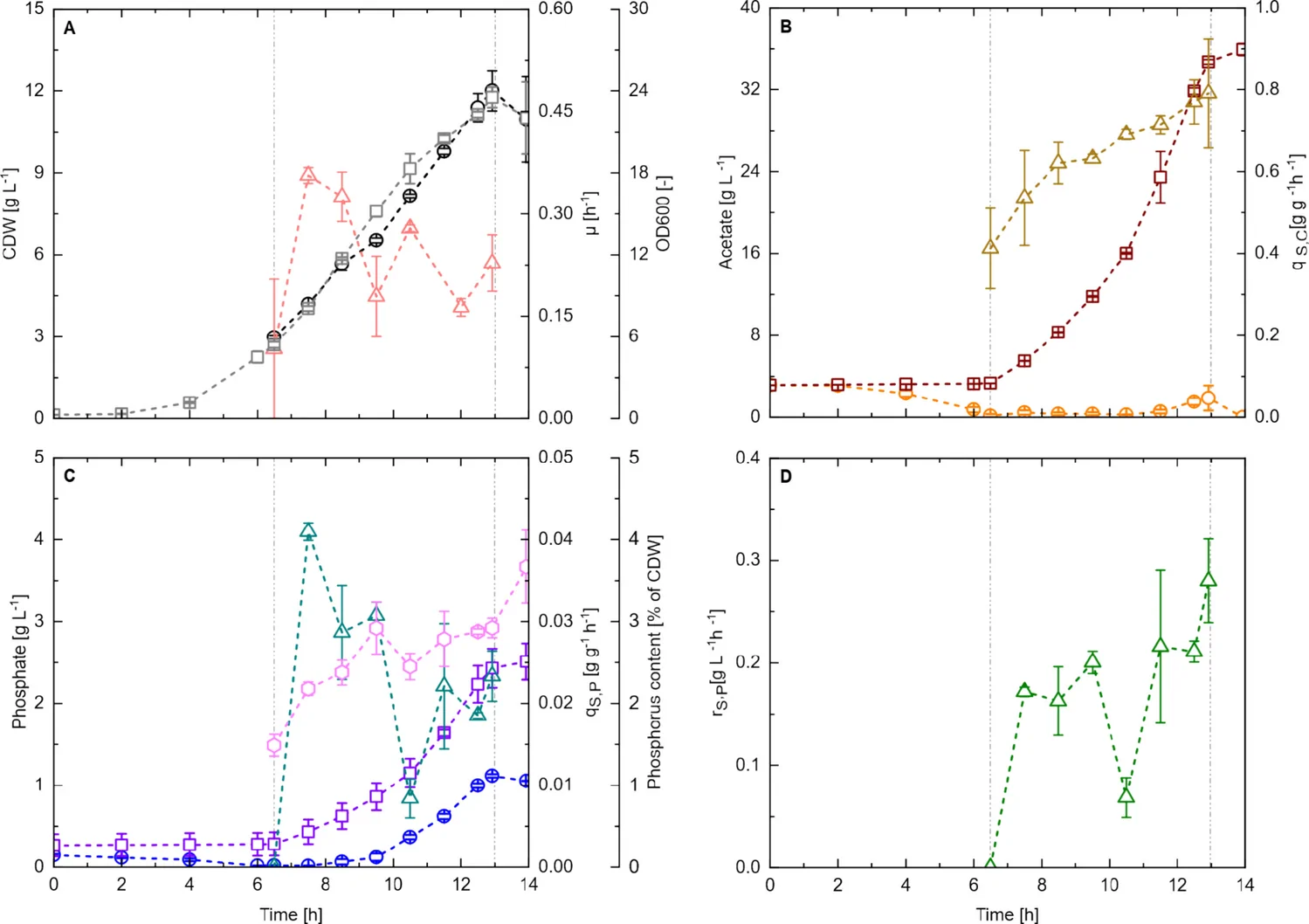
Batch/fed-batch bioreactor cultivations with *A. tjernbergiae* with an increased phosphate concentration in the feed solution. The start of the exponential feed (*μ*_set_ = 0.3 h^−1^) is marked with the dotted line at 6.5 h, and the feed stop at 13 h. The error bars indicate ± SD. Transient foaming led to short-term biomass loss and artificial distortion of *μ*. Therefore, an interpolated *μ* value at 12 h is shown. **A** CDW (black circles), specific growth rate *μ* (red triangles), and OD_600_ (grey squares). **B** Acetate concentration (orange circles), total acetate addition (red squares), and specific acetate uptake rate (golden triangle). **C** Phosphate concentration (blue circles), total phosphate addition (violet squares), specific phosphate uptake rate (cyan triangles), and specific phosphorus content (pink circles). **D** Volumetric phosphate uptake rate

Increasing the phosphate supply during exponential feeding increased biomass formation and total phosphate uptake (Fig. 4). A cumulative phosphate uptake of 1.2 ± 0.0 g L^−1^ was achieved within 13.9 h of cultivation. The biomass concentration increased exponentially until 8.5 h, after which growth rates declined, reaching a maximum CDW of 12.0 ± 0.7 g L^−1^ at the end of the feed phase. Acetate was not growth-limiting throughout the cultivation, and q_S,C_ increased from 0.41 ± 0.01 g (g h)^−1^ to 0.79 ± 0.13 g (g h)^−1^ for 6.5–12.9 h; q_S,P_ peaked immediately after the batch phase at 41.0 ± 1.1 mg (g h)^−1^ and subsequently stabilised at approximately 30 mg (g h)^−1^ until 9.5 h. Y_XS,P_ decreased from 11.3 ± 2.0 to 8.5 ± 0.3 g g^−1^ during the same period, while Y_PS,C_ declined from 38.8 ± 2.3 to 27.3 ± 0.9 mg g^−1^ (Table 1). The highest volumetric phosphate uptake rate r_S,P_ = 280.2 ± 40.8 mg (L h)^−1^ was observed during the final hour of feeding. The specific phosphorus content rose until 9.5 h, stabilising at approximately 3% (w w^−1^) thereafter. These observations prove that the exponential feeding regime with elevated phosphate supply achieves a higher final biomass while further increasing q_S,P_ and r_S,P_.

**Table 1:** Comparison of growth parameters, uptake rates, and yields for the two bioreactor experiments with different phosphate concentrations in the feed solutions

	C:P 100:1 (with P-pulses)	C:P 100:2
Exponential growth phase (h)	6.5–10.5	6.5–12.9
Max. biomass (g L^−1^)	8.6 ± 0.4 (10.5 h)	12.0 ± 0.7 (12.9 h)
*µ*_max_ (h^−1^)	0.28 ± 0.00 (10 h)	0.36 ± 0.01 (6.5–10.5 h) 0.28 ± 0.00 (10.5–12.9 h)
q_S,Pmax_ (mg (g h)^−1^)	31.2 ± 0.9 (10.5 h)	41 ± 1.1 (7.5 h)
q_S,P_ (mg (g h)^−1^)	22.5 ± 2.7	23.8 ± 0.9 (6.5–10.5 h) 20.0 ± 1.8 (10.5–12.9 h)
q_S,Cmax_ (g (g h)^−1^)	0.76 ± 0.09 (10.5 h)	0.79 ± 0.13 (12.9 h)
q_S,C_ (g (g h)^−1^)	0.63 ± 0.03	0.63 ± 0.01 (6.5–10.5 h) 0.73 ± 0.04 (10.5–12.9 h)
q_S,N_ (mg (g h)^−1^)	N/A	30.0 ± 1.5 (6.5–10.5 h) 30.4 ± 3.3 (10.5–12.9 h)
Y_XS,C_ (g g^−1^)	0.39 ± 0.03	0.43 ± 0.0 (6.5–10.5 h) 0.23 ± 0.02 (10.5–12.9 h)
Y_XS,N_ (g g^−1^)	N/A	9.0 ± 0.7 (6.5–10.5 h) 5.4 ± 0.6 (10.5–12.9 h)
Y_XS,P_ (g g^−1^)	11.3 ± 1.3	11.3 ± 2 (6.5–10.5 h) 8.5 ± 0.3 (10.5–12.9 h)
Y_PS,C_ (mg g^−1^)	35.0 ± 1.5	38.8 ± 2.3 (6.5–10.5 h) 27.3 ± 0.9 (10.5–12.9 h)
Max. specific phosphorus content (%-w w^−1^)	2.7 ± 0.4 (11 h) 2.81 ± 0.0 (13 h)	2.9 ± 0.1 (12.9 h) 3.6 ± 0.4 (13.9 h)

## Discussion

The results of this study demonstrate that iron availability is a critical factor for the growth and, therefore, the phosphate uptake of *A. tjernbergiae*. Iron salt additions increasing its concentration between 1 and 8 mgL^−1^ in shake flask experiments resulted in a six-fold increase in optical density compared to the reference medium containing only 0.1 mgL^−1^ iron salt, indicating that iron was strongly growth-limiting under the reference conditions. When normalised to biomass, these additions correspond to 0.55–4 mg iron g^−1^ biomass, defining a range that appears beneficial for growth. The supplementation of 1.6 mg iron g^−1^ biomass enabled the formation of 12.0 ± 0.7 g L^−1^ biomass in 12.9 h in the bioreactor cultivations with an increased amount of phosphate in the feed. This is three-fold higher than the biomass previously reported for *Acinetobacter* spp. under comparable conditions, where 4 g L^−1^ was achieved after 12 h of cultivation on acetate and ammonium (Srivastava and Srivastava 2005). To date, no iron-dependent metabolic pathways unique to *Acinetobacter* spp. have been described. However, iron functions as an essential co-factor in the electron transport chain and numerous other enzymatic reactions, making it essential for respiration, the central energy metabolism, and other processes (Eijkelkamp et al. 2011; Frawley and Fang 2014). Since polyP synthesis is ATP-dependent and ATP generation relies on the respiratory activity (Van Veen et al. 1993; Trelstad et al. 1999), we hypothesise that the increased iron requirement of *A. tjernbergiae* is linked to its respiratory capacity and therefore phosphate assimilation activity. Iron availability supports the synthesis of iron-dependent proteins in the electron transport chain, which enhances the proton-motive force and ATP production. Since phosphate transport into the cell is driven by this proton gradient, and since polyP polymerisation requires ATP (Van Veen et al. 1994; Itoh and Shiba 2004), a robust respiratory infrastructure is a pre-requisite for active phosphate uptake. In contrast to iron, supplementation with zinc did not lead to any detectable growth improvements, suggesting that the zinc concentration in the PR medium was already sufficient to meet cellular demands.

In other *Acinetobacter* spp. such as *A. baumannii*, enhanced growth under iron-replete conditions has been attributed to reduced synthesis of iron-chelating siderophores (Eijkelkamp et al. 2011; Sheldon and Skaar 2020). Siderophores are a chemically diverse group of secondary metabolites with a typically high affinity for iron. As siderophore production imposes an energetic burden, increased iron availability may lead to the redirection of resources from iron accessibility to biomass formation. Specifically, the biosynthesis and secretion of siderophores require significant amounts of cellular ATP and metabolic precursors (Frawley and Fang 2014), a process that is tightly regulated by iron-dependent repressors in *Acinetobacter* spp. (Sheldon and Skaar 2020; Zhang et al. 2025). When iron is supplied in excess, the metabolic repression of these pathways would allow *A. tjernbergiae* to utilise more energy for growth. If *A. tjernbergiae* produces siderophores, this trait could also be relevant for phosphorus recovery in wastewater treatment systems, particularly where chemical phosphorus removal via iron precipitation is applied. In anaerobically digested sludge from such facilities, 40–70% of total phosphorus has been reported to be associated with iron-phosphate complexes (Wilfert et al. 2016; Zhang et al. 2019; Wang et al. 2021). Although siderophore systems have not been specifically characterised in *A. tjernbergiae*, genome sequencing data are available for the type strain, enabling in silico identification of iron acquisition genes. Moreover, iron uptake mechanisms, including siderophore-mediated systems, are widely conserved across the genus *Acinetobacter* (Zhang et al. 2025), and mobile genetic elements encoding such functions have been identified in multiple species (Hamidian 2022). This suggests that related iron acquisition systems are likely present in *A. tjernbergiae*. However, siderophore production is not universal among *Acinetobacter* spp., and siderophore-mediated mobilisation of phosphorus from iron-bound fractions in WWTP has not yet been investigated (Zhao et al. 2023). For other systems and microorganisms, siderophores have been shown to facilitate the mobilisation of phosphorus (Ghosh et al. 2015; Romano et al. 2017; Wang et al. 2021).

Consistent with previous studies on *Acinetobacter* spp., acetate and ammonium were confirmed to be suitable carbon and nitrogen sources for *A. tjernbergiae*, supporting both high growth rates and biomass accumulation (Chen et al. 2021; Han et al. 2018; Srivastava and Srivastava 2005; Van Groenestijn et al. 1989). In shake flask studies with *A. junii* strain LH4, acetate outperformed several alternative carbon sources like propionate, succinate, citrate, glucose, and sucrose. Ammonium was superior to the nitrogen sources nitrate, nitrite, and peptone (Han et al. 2018). It should be noted that growth on acetate is strongly pH-dependent. In experiments with *A. johnsonii*, no growth occurred below pH 6, while growth rates increased strongly up to pH 7 and peaked at pH 9 (Van Groenestijn et al. 1989). Therefore, it is assumed that the pH of 7.5 as applied in bioreactor cultivations in this study is within a range suitable for acetate assimilation. An optimal molar C:N ratio of about 9:1 was identified, which is consistent with values reported for other *Acinetobacter* spp. (Pauli and Kaitala 1995; Han et al. 2018; Chen et al. 2021). Similar to *A. junii* LH4, which exhibits limited growth on peptone, *A. tjernbergiae* appears unable to assimilate all complex nitrogen sources provided by tryptone and yeast extract (Han et al. 2018). This is supported by the lower final OD observed when using tryptone in this study. Results from nitrogen-limited bioreactor cultivations also showed substantially lower growth and final biomass concentration (data not shown).

The growth rate decline in the increased phosphate feeding rate experiment after 8.5 h was accompanied by reduced yield coefficients. While extended fed-batch processes inherently impose physiological stress and culture variability, nutrient limitation cannot be excluded. Especially magnesium, the main polyP counterion, might become limiting under certain conditions, as literature reports a wide range of 0.39–1.41 g magnesium per 1 g of phosphorus in media (Van Groenestijn et al. 1988; Pauli and Kaitala 1995). It is, however, doubtful if a long-lasting fed-batch operation is of relevance, as any application would rather require a continuous operation with cell retention due to overall rather low concentrations in the wastewater feed fractions. Then, batch and fed-batch would be the modes of choice only for an initial cultivation phase.

Across all bioreactor experiments, a strong coupling between q_S,C_, growth, and q_S,P_ was observed. Phosphate assimilation was most pronounced during active growth and periods of excess energy availability, consistent with previous reports for polyP-accumulating *Acinetobacter* spp. (Ohtake et al. 1985; Van Groenestijn 1988; Ghigliazza et al. 1998). Results further confirm that *A. tjernbergiae* performs luxury phosphate uptake. At a comparable specific growth rate of *µ* = 0.3 h⁻^1^, higher q_S,P_ values were observed when phosphate concentrations were increased, as demonstrated by the phosphate pulse, and the increased C:P ratio of 100:2 in the respective bioreactor experiments. These observations indicate that *A. tjernbergiae* accumulates polyP as an energy reservoir under the applied conditions (Ghigliazza et al. 1998). Results also highlight that a C:P ratio of 100:1 does not limit *A. tjernbergiae* growth, while it does limit phosphate uptake.

The q_S,P_ profile at increased phosphate feeding further demonstrates that *A. tjernbergiae* performs the overplus phenomenon, as has been discussed for other *Acinetobacter* spp. (Ohtake et al. 1985; Pauli and Kaitala 1997; Van Niel et al. 1999) and polyP-storing organisms (Plouviez et al. 2025). The maximum q_S,P_ value of 41.0 ± 1.1 mg (g h)^−1^ occurred immediately following the phosphorus-limited batch phase, whereas q_S,P_ values dropped under prolonged cultivation at phosphate excess. This uptake profile is characteristic of the overplus phenomenon, which occurs when phosphate-deprived cells are transferred to phosphate-rich media (Ohtake et al. 1985). A proposed metabolic explanation is that once sufficient intracellular polyP has accumulated, the expression of PPK1, responsible for polyP formation, is downregulated, reducing further polyP synthesis and phosphate uptake (Trelstad et al. 1999).

Consistent with other studies on *Acinetobacter* spp., the addition of a carbon source to the carbon-starved culture resulted in the immediate resumption of growth and phosphate uptake (Ohtake et al. 1985; Van Groenestijn and Deinema 1987). It remains unclear from the results of this study to what extent this adaptability is supported by energy production from polyP breakdown and how long the starvation phase can be extended. Previous studies report ATP generation from polyP degradation and instantaneous growth recovery after starvation periods of up to 10 h (Ohtake et al. 1985; Van Groenestijn and Deinema 1987).

Exceptionally high phosphate assimilation rates were achieved in this study. The q_S,Pmax_ of 41.0 ± 1.1 mg (g h)^−1^ ranks among the highest reported for *Acinetobacter* spp. under overplus conditions (Hao and Chang 1987; Pauli and Kaitala 1997). Sustained q_S,P_ values of 30 mg (g h)^−1^ are at the upper range of published data gained at comparable process conditions (Table 2). The highest total phosphate assimilation rate of 180 mg (L h)^−1^ exceeds literature values by more than six-fold. For comparison, Van Groenestijn et al. (1989) reported a maximum phosphate assimilation of 27.6 mg (L h)^−1^ in an 8 h batch experiment with 1.15 g L^−1^ biomass and an intracellular phosphorus content of 6% (w w^−1^).

**Table 2:** Comparison of results from the increased phosphate feeding rate experiment and selected results from studies with pure cultures of *Acinetobacter* spp.

Strain	Cultivation time	Maximum biomass	Spec. PO_4_ assimilation rate^b^	Spec. phosphorus content	Spec. polyP content^e^	Reference
Unit	h	g L^−1^	mg (g h)^−1^	% (w w^−1^)	% (w w^−1^)	
Unclassified strain 603	4	0.3	40^c^	4.8^d^	14.4^c^	Hao and Chang 1987
*A. johnsonii* 210A	8	1.2	23^c^	6	15.1^c^	Van Groenestijn et al. 1989
*A. Iwofii* 9003^a^	11	0.38	Initial: 36 Average: 6.4^c^	2.1^d^	5.3^c^	Pauli and Kaitala 1997
*A. calcoaceticus* NRRLB-552	12	4	3.1^c^	1.2^c^	3^c^	Srivastava and Srivastava 2005
*A. calcoaceticus* IAM 12087	20	0.7	8.8^c^	3.4^d^	10^c^	Ohtake et al. 1985
Unclassified	64	0.6	2.9^c^	7.9^c^	20	Deinema et al. 1980
*A. tjernbergiae* 7N16	14	12	Max: 40 Average: 15	3	7.6	This study

All experiments were performed in batches

^a^Preculture in phosphate-limited medium, ^b^mg PO_4_ g^−1^ biomass h^−1^, ^c^own calculation based on PO_4_ uptake per biomass in the respective publication. ^d^Molecular weight ratios used to convert PO_4_, P, and PO_3_. ^e^polyP as PO_3_ units. It is assumed that all phosphorus uptake is stored as polyP

The polyP fraction can be estimated when considering reports from others. Literature values indicate that total phosphorus varies from 0.93 to 5.3% (w w^−1^) of the cell dry mass (Ohtake et al. 1985). During logarithmic growth across four different strains of *A. calcoaceticus*, orthophosphate accounted for 7–10% (w w^−1^) of total cellular phosphorus, while polyP constituted 40–60% (w w^−1^). The polyP fraction may further increase to approximately 70% (w w^−1^) of total cellular phosphorus when present in excess (Ohtake et al. 1985).

Despite comparably high growth and phosphate assimilation rates, intracellular phosphorus contents remained relatively modest at 2–3.6% (w w^−1^) across bioreactor experiments. Although these values exceed the specific phosphorus content of 1.2–2.1% (w w^−1^) reported in some batch cultivation studies, values are substantially below 6–8% (w w^−1^) reported for cultivations with other strains (Table 2). However, many of these studies employed nutrient limitation to maximise intracellular phosphorus accumulation at the expense of biomass formation. Carbon or sulphur limitation has been reported as particularly advantageous in this regard (Ohtake et al. 1985; Van Groenestijn et al. 1989; Weltin et al. 1996). Phosphorus associated with typical microbial growth accounts for approximately 1.5–2% (w w^−1^) of dry biomass. polyP accumulation can increase this value substantially. Individual microalgal isolates accumulated up to 14% (w w^−1^) polyP, equivalent to 5.4% (w w^−1^) phosphorus, whereas in *Saccharomyces cerevisiae* VH2.200, 7.4% (w w^−1^) phosphorus accumulation is reported (Schaedig et al. 2023; Christ and Blank 2019). Cellular phosphorus contents exceeding 10% (w w^−1^) have been reported for *Microlunatus phosphovorus* NM-1, while *Acinetobacter* strain 210 A reached up to 10% (w w^−1^) (Nakamura et al. 1995; Van Groenestijn et al. 1989). By contrast, non-engineered *Escherichia coli* MV1184 contained only approximately 1.8% (w w^−1^) phosphorus, whereas recombinant derivatives reached transient maxima of 16% (w w^−1^) (Kato et al. 1993). Intracellular phosphorus contents of 8–10% (w w^−1^) in axenic cultures of *Acinetobacter* spp. are unique among natural isolates (Deinema et al. 1980; Van Groenestijn et al. 1989). In non-axenic cultures enriched with PAOs, a phosphorus content of 8–15% (w w^−1^) has been measured in activated sludge (Blackall et al. 2002; Yang et al. 2017).

## Outlook

The current study identified key factors influencing growth and phosphate uptake in *A. tjernbergiae*, with increased iron addition enabling unprecedented biomass concentrations and volumetric phosphate uptake rates. These results position *A. tjernbergiae* as a promising platform for microbial phosphate recovery from wastewater streams, among other microorganisms, which might not have the same ability to survive in and adapt to environmental conditions as quickly as *Acinetobacter* spp. To achieve better phosphate removal, process optimisation should substantially increase intracellular phosphorus accumulation, enhancing q_s_P and overall process efficiency. Maximum volumetric phosphate uptake should thus be achievable via two process strategies: either a two-stage process with a separate growth stage and a separate phosphate uptake stage at low nutrient availability, or direct growth in phosphorus-rich medium, while maintaining a balance between biomass accumulation and volumetric phosphate assimilation. A co-feeding strategy that supplies additional carbon might be suitable to increase the biomass concentration and cope with otherwise low-carbon concentrated wastewater side streams in a continuous operation with cell retention. Perfusion has also been shown to be applicable to low-cost processes with high liquid turnover. It has the potential to allow the integration of a microbial phosphate removal step into a wastewater treatment concept in the future.

## List of parameters

**Table:** 

i	C, acetate; N, nitrogen; P, phosphate	^−^
μ	Specific growth rate	h^−1^
qs	Specific substrate uptake rate	g (gh)^−1^
rs	Volumetric substrate uptake rate	g (Lh)^−1^
Si	Substrate concentration	g L^−1^
t	Cultivation time	h
V	Reactor filling volume	L
X	Biomass concentration	g L^−1^
XV	Average of X×V	g
$Y_{XS,i}$	Biomass yield on substrate *i*	g g^−1^
$Y_{PS,C}$	Yield of phosphate on acetate uptake	mg g^−1^

## Data Availability

The data that support the findings of this study are available from the corresponding author upon a reasonable request.

## Declarations

During the preparation of this work, the authors used the generative AI technologies ChatGPT (GPT-4, OpenAI, USA) and Perplexity AI (Perplexity AI, USA) to improve language and readability. After using these tools, the authors reviewed and edited the content as needed and take full responsibility for the content of the publication.
